# Elevated Triggering Receptor Expressed on Myeloid Cells 2 Expression in Tumor-Associated Macrophages Suppresses Cytotoxic T Cell Infiltration and Facilitates Immune Escape in Colorectal Cancer

**DOI:** 10.1016/j.jcmgh.2026.101842

**Published:** 2026-06-30

**Authors:** Xixi Liu, Xiaohan Yuan, Lijie Ge, Haimei Sun, Lili Zhong, Aowei Qin, Yuxue Guan, Tingyi Sun, Deshan Zhou, Shu Yang, Bo Wu

**Affiliations:** 1 Department of Histology and Embryology, School of Basic Medical Sciences, Capital Medical University, Beijing, China; 2 Beijing Key Laboratory of Cancer Invasion and Metastasis Research, Beijing, China; 3 Capital Center for Children's Health, Capital Medical University, Capital Institute of Pediatrics, Beijing, China

**Keywords:** Cancer-Associated Fibroblasts, Colorectal Cancer, Immune Escape, TREM2, Tumor-Associated Macrophage, Tumor Microenvironment

## Abstract

**Background & Aims:**

Emerging evidence supports a crucial role for tumor-associated macrophages in shaping the immunosuppressive tumor microenvironment. Furthermore, research has identified that the triggering receptor expressed on myeloid cells 2 has immunomodulatory functions. The present investigated the potential effect of triggering receptor expressed on myeloid cells 2 expression in tumor-associated macrophages on facilitating immune evasion in colorectal cancer.

**Methods:**

Immunohistochemical analysis of clinical specimens, complemented by extensive data mining from The Cancer Genome Atlas, revealed a significant upregulation of triggering receptor expressed on myeloid cells 2 in colorectal cancer-associated tumor-associated macrophages, with this upregulation exhibiting a correlation with poor patient prognosis.

**Results:**

Mechanistically, triggering receptor expressed on myeloid cells 2^+^ tumor-associated macrophages were found to drive fibroblast activation through transforming growth factor-β signaling, inducing fibroblast-activated protein–positive cancer-associated fibroblasts that secrete collagen I/III to establish dense peritumoral barriers. Spatial profiling revealed that these fibrous structures physically impede CD8^+^ T-cell infiltration, restricting cytotoxic lymphocytes to stromal compartments. Intriguingly, triggering receptor expressed on myeloid cells 2 deficiency enhanced the secretion of matrix metalloproteinase 13 by macrophages, thereby promoting extracellular matrix degradation and improving T-cell penetration. In vivo, *Trem2*-knockout mice showed a reduction in tumor growth with enhanced intratumoral CD8^+^ T-cell infiltration compared with wild-type controls.

**Conclusions:**

Our findings establish triggering receptor expressed on myeloid cells 2^+^ tumor-associated macrophages as central regulators of stromal remodeling and suggest that therapeutic targeting of the triggering receptor expressed on myeloid cells 2/transforming growth factor-β/fibroblast-activated protein pathway may overcome immune resistance in patients with colorectal cancer.

## Graphical abstract

**Figure:**
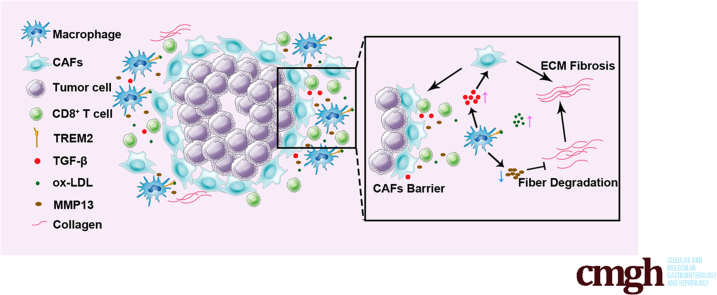

**Summary**

In colorectal cancer, TREM2⁺ macrophages secrete transforming growth factor-β to activate cancer-associated fibroblasts, forming a stromal barrier that physically excludes CD8⁺ T cells.

**What You Need to Know**

**Background**

Triggering receptor expressed on myeloid cells 2^+^ tumor-associated macrophages are upregulated in colorectal cancer and correlate with poor prognosis. This study aimed to elucidate how triggering receptor expressed on myeloid cells 2 drives cancer-associated fibroblast activation to form collagen barriers that exclude CD8^+^ T cells.

**Impact**

This study identifies triggering receptor expressed on myeloid cells 2+ tumor-associated macrophages as key regulators of immune-exclusive stromal remodeling. Targeting the triggering receptor expressed on myeloid cells 2/transforming growth factor-β/fibroblast-activated protein axis holds potential to overcome immunotherapy resistance by restoring T-cell infiltration and extracellular matrix degradation.

**Future Directions**

Future studies should validate the triggering receptor expressed on myeloid cells 2/transforming growth factor-β/fibroblast-activated protein pathway as a therapeutic target in preclinical colorectal cancer models and explore their combination with immune checkpoint inhibitors to enhance antitumor immunity and improve patient outcomes.

The latest cancer statistics report released by the American Cancer Society reported that colorectal cancer (CRC) now ranks third in terms of new cases and is a leading cause of cancer-related deaths.^1^^,^^2^ A high incidence of local and distant metastases, along with disease relapse, contribute to the poor prognosis associated with CRC. Over the past 2 decades, immunotherapy has emerged as a prominent therapeutic approach in the field of clinical oncology. Immunotherapy represents a novel form of cancer treatment that has shown promise in the elimination of a range of solid tumors via a mechanism that differs from conventional chemotherapy or targeted therapy.^3^ However, at present, its benefits are restricted to a select group of patients. The fundamental principle of immunotherapy is to restore the balance between the immune system and tumor cells by enhancing immune function.^4^ The tumor microenvironment (TME), defined as the area immediately surrounding tumor cells, is a critical determinant in suppressing immune cell function.^5^^,^^6^ The presence of a robust extracellular matrix (ECM) within the TME plays a key role, as it contributes to the development of drug resistance. Targeting the ECM can be considered a novel therapeutic approach.^7^ Although previous studies have concentrated on the interactions among immune cells within the TME, the mechanisms by which tumors evade immune control by establishing an immune-privileged microenvironment remain elusive.

A member of the immunoglobulin superfamily, triggering receptor expressed on myeloid cells 2 (TREM2), is a transmembrane receptor predominantly expressed in myeloid cells such as macrophages. It features an extracellular V-type immunoglobulin domain, a transmembrane domain, and a short cytoplasmic tail. Emerging research indicates that TREM2 is expressed in various cancers where it exerts immunomodulatory functions.^8^

Multiple studies have provided evidence for an immunosuppressive role of TREM2 in cancers. However, the potential of TREM2 expression in tumor-associated macrophages (TAMs) to regulate the activation of cancer-associated fibroblasts (CAFs), thereby facilitating immune escape, remains unknown. The present study investigated the effects of TREM2 expression in TAMS within the TME of CRC to determine whether and how it plays an immunosuppressive role in this environment. The experiments conducted in this study demonstrated that TREM2 is capable of activating CAFs, thereby influencing their spatial distribution. Furthermore, TREM2 was identified as a regulator of the secretion of matrix metalloproteinase 13 (MMP13) by macrophages, which consequently affects collagen degradation. Consequently, the activated fibroblast-activated protein (FAP)^+^ CAFs, in conjunction with stromal fibers, form a compact physical barrier that impedes the interaction between CD8^+^ T cells and tumor cells. Furthermore, the extensive sheet-like distribution of collagen in the stroma may hinder the migration of CD8^+^ T cells.

## Results

### Triggering Receptor Expressed on Myeloid Cells 2 Expression on Tumor-Associated Macrophages Is Elevated Within Colorectal Cancer

**Figure 1:**
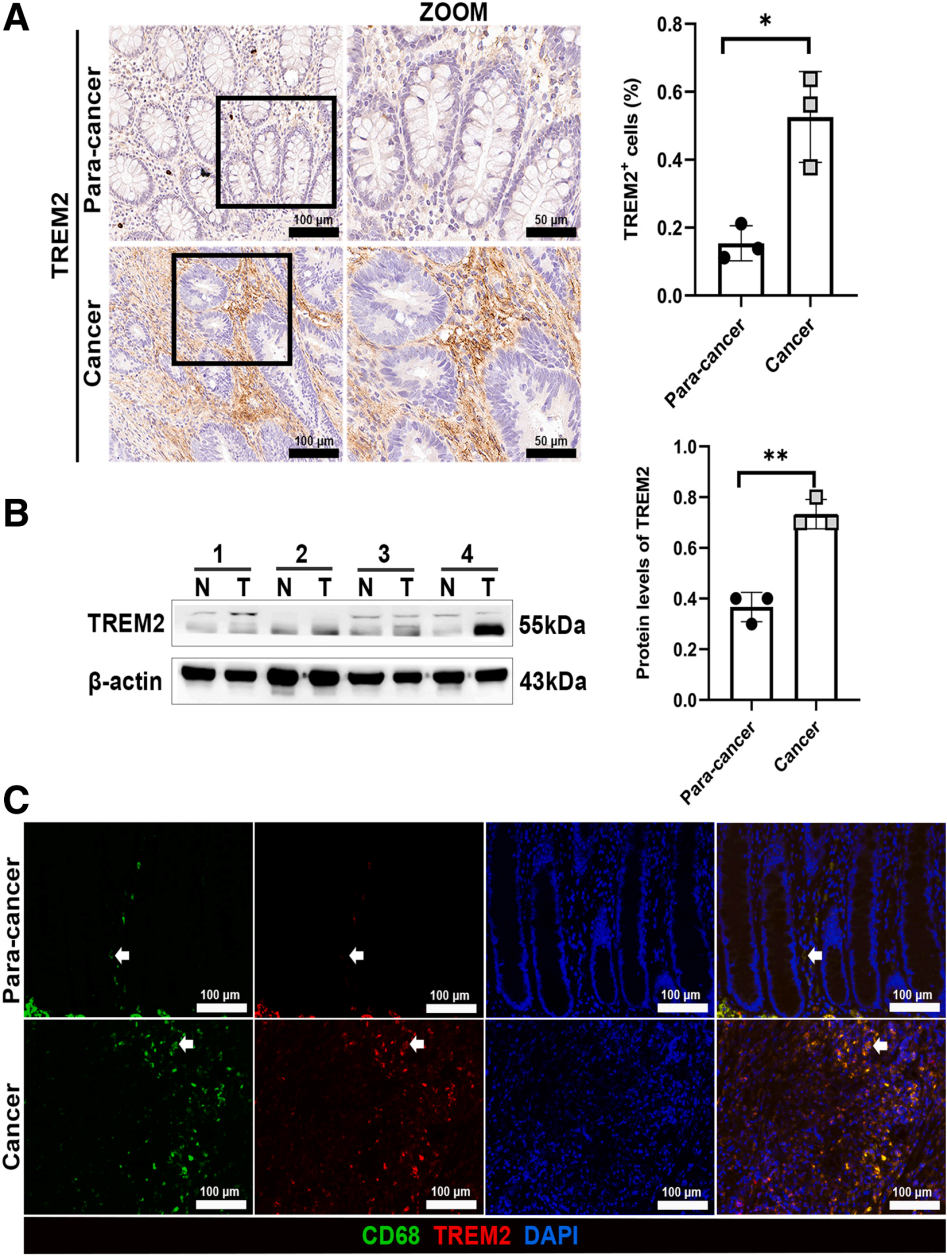
**Elevated TREM2 expression by TAMs in CRC.** (*A*) Immunohistochemical staining of TREM2 in CRC (*brown*). Scale bars, 100 μm (*left row*); 50 μm (*right row*). (*B*) Western blotting of TREM2 in CRC. N, para-cancer; T, cancer. (*C*) Immunofluorescence staining illustrates colocalization of CD68 (*green*) and TREM2 (*red*) in CRC tissues. The *arrows* indicate macrophages. Scale bars, 100 μm. ∗*P* < .05; ∗∗*P* < .01. DAPI, 4′,6-diamidino-2-phenylindole.

To gain insight into the role of TREM2 in CRC, we examined TREM2 expression in clinical specimens from patients with CRC. Immunohistochemical staining was employed to observe the expression patterns of TREM2 within CRC tissues. Compared with its expression in the adjacent noncancerous tissue, TREM2 was found to be highly expressed in the tumor samples and mainly expressed in the stromal area rather than on the tumor cells (Figure 1*A*). These results were subsequently confirmed by Western blotting (Figure 1*B*). In view of the fact that TREM2 is generally expressed on myeloid cells, we observed the spatial relationship between TREM2 and the macrophage marker CD68 in CRC. Immunofluorescence staining revealed colocalization of CD68 with TREM2, suggesting that TREM2 was expressed in TAMs at a significantly higher level than in the adjacent tissues (Figure 1*C*). These results suggest a potential role of TREM2 expressed in TAMs in the TME of CRC.

### Triggering Receptor Expressed on Myeloid Cells 2 Is Associated With Fibrous Matrix in Colorectal Cancer

**Figure 2:**
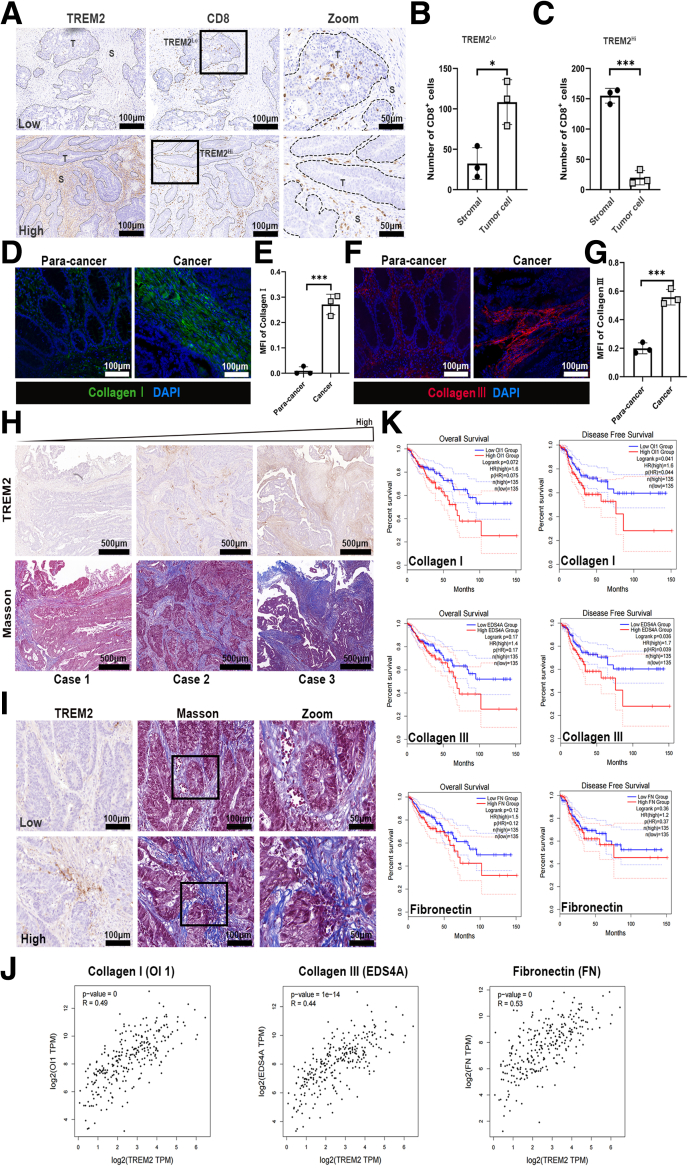
**Correlation of TREM2 with the fibrous matrix in CRC.** (*A–C*) Immunohistochemical staining for TREM2 and CD8 in CRC tissues. S, stroma; T, tumor cell. Scale bars, 100 μm and 50 μm (magnified images, marked by *black boxes*). (*D–G*) Immunofluorescence staining of collagen I (*green*) and collagen III (*red*) in CRC tissues. Scale bars, 100 μm. (*H* and *I*) Immunohistochemical staining of TREM2 in conjunction with Masson’s Trichrome staining of collagen fibers. Representative images from 3 patients with CRC (*H*), and distinct areas of a single patient with CRC (*I*). Scale bars, 500 μm (*H*), 100 μm (*I*), and 50 μm (*I*, magnified images, marked by *black boxes*). (*J*) Analysis of the correlation between TREM2 and collagen I (OI 1), collagen III (EDS4A), and fibronectin (FN). (*K*) Kaplan–Meier analysis of CRC patient survival in relation to collagen I (OI 1), collagen III (EDS4A), and fibronectin (FN) expression. Expression levels above 50% were categorized as “high expression,” and those below 50% were considered “low expression.” ∗*P* < .05; ∗∗∗*P* < .001. DAPI, 4′,6-diamidino-2-phenylindole.

**Figure 3:**
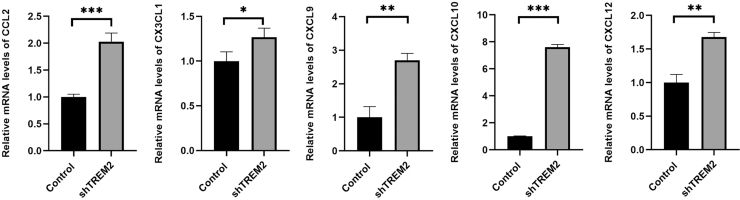
**Regulation of chemokines by TREM2 expression on macrophages.** CCL2, CX3CL1, CXCL9, CXCL10 and CXCL12 messenger RNA (mRNA) expression in macrophages. ∗*P* < .05; ∗∗*P* < .01; ∗∗∗*P* < .001. sh, short hairpin.

Previous research suggested TREM2 as a factor with tumor-promoting properties in CRC,^8^ but the precise mechanism by which TREM2 promotes CRC remains unclear. In this study, we investigated the potential influence of TREM2 on the distribution of CD8^+^ T cells in CRC tissues. Immunohistochemical staining of human CRC tissues showed that CD8^+^ T cells were predominantly distributed in the stromal areas with higher TREM2 expression instead of among the tumor cell clusters (Figure 2*A–C*). Conversely, in the area with lower TREM2 expression, a considerable presence of CD8^+^ T cells was observed among the tumor cells, which may potentially allow direct contact with them (Figure 2*A–C*). This distribution pattern suggests that TREM2 may regulate the infiltration of CD8^+^ T cells within the TME. To explore the way in which TREM2 affects CD8^+^ T-cell infiltration, we detected the expression of macrophage-derived chemokines known to be pivotal in regulating CD8^+^ T-cell migration and distribution, in the context of TREM2 expression. The results showed no significant positive correlation between the stromal accumulation of CD8^+^ T cells and the levels of these chemokines (Figure 3), suggesting that alternative mechanisms may be involved in the regulation of CD8^+^ T-cell infiltration by TREM2.

It has been postulated that the collagen components present within the tumor stroma may impede the penetration of antitumor drugs and diminish immune cell infiltration.^9^ Therefore, the collagen content in CRC samples was investigated by immunofluorescence staining, which demonstrated significantly higher levels of collagen I and III in CRC tissues than in adjacent noncancerous tissues (Figure 2*D–G*). Masson’s Trichrome staining further revealed an increase in collagen content within tumor tissues with increasing levels of TREM2 (Figure 2*H*). A positive correlation between collagen content and TREM2 expression was identified in distinct areas of a single CRC sample (Figure 2*I*). Analysis of data from The Cancer Genome Atlas (TGCA) database also revealed a positive correlation between TREM2 and fibrous matrix components, including fibronectin, collagen I, and collagen III (Figure 2*J*). Furthermore, survival analysis using the GEPIA2 platform demonstrated that patients with CRC and higher expression of collagen I, collagen III, and fibronectin had significantly shorter overall survival and disease-free survival (Figure 2*K*), indicating the potential prognostic significance of TREM2-associated fibrous matrix in CRC.

### Triggering Receptor Expressed on Myeloid Cells 2 Activates Cancer-Associated Fibroblasts in Tumor Stroma

**Figure 4:**
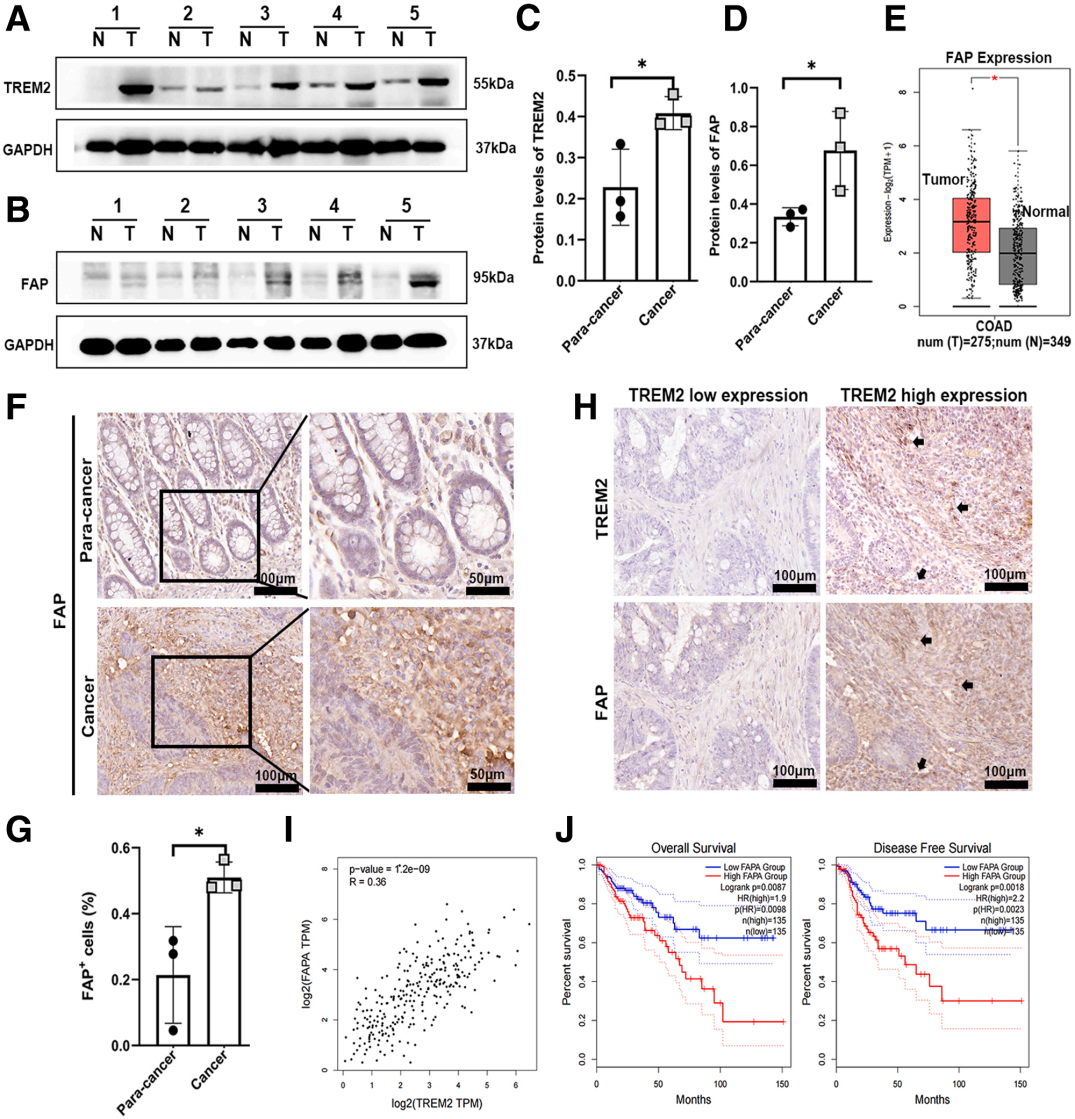
**Correlation of TREM2 with the fibrous matrix in CRC.** (*A–D*) Western blot analysis of FAP protein expression in CRC. N, para-cancer tissue; T, cancer tissue. (*E*) Kaplan–Meier analysis of The Cancer Genome Atlas (TCGA) data, showing the prognostic significance of FAP expression in colon adenocarcinoma (COAD). Tumor color, *red*; normal color, *gray*. (*F* and *G*) Immunohistochemical staining of FAP in CRC tissues. ∗*P* < .05. Scale bars, 100 μm (*left row*), 50 μm (*right row*). (*H*) Immunohistochemical staining of FAP and TREM2 in CRC tissues. Scale bars, 100 μm. (*I*) Kaplan-Meier analysis of TCGA data, examining the correlation between FAP and TREM2 expression. (*J*) Kaplan–Meier analysis of CRC patient survival using TCGA data, illustrating the correlation between FAP expression and both overall survival and disease-free survival. Expression is categorized based on a threshold of more than 50% (high expression) vs below 50% (low expression).

In light of the findings that TREM2 may orchestrate the fibrous matrix in CRC, an investigation was conducted into the changes in collagen-producing CAFs. The TME is rich in a diverse range of CAF subtypes with distinct surface markers. FAP, a marker commonly used to identify CAFs in tumors, exhibited a significant increase in human CRC tissues compared with adjacent noncancerous tissues, as demonstrated by Western blotting (Figure 4*A–D*) and immunohistochemical staining (Figure 4*F* and *G*). This observation was consistent with the data obtained from The Cancer Genome Atlas database (Figure 4*E*). Immunohistochemical analysis of TREM2 and FAP expression on serial tissue sections showed that the stromal areas with low TREM2 levels had sparse FAP expression, whereas those with higher TREM2 levels exhibited intensive FAP expression (Figure 4*H*). Analysis of The Cancer Genome Atlas data showed that the level of FAP expression was significantly correlated with TREM2 expression (Figure 4*I*), indicating that TREM2 may increase collagen production in the tumor stroma by activating CAFs. Furthermore, higher FAP expression was associated with shorter overall survival and disease-free survival (Figure 4*J*). The findings indicate that macrophages with elevated TREM2 expression may be a key driver of the development of the fibrous matrix within the CRC stroma, through the stimulation of FAP^+^ CAFs.

### Fibroblast-Activated Protein^+^ Cancer-Associated Fibroblasts Impede CD8^+^ T-Cell Infiltration in Colorectal Cancer

**Figure 5:**
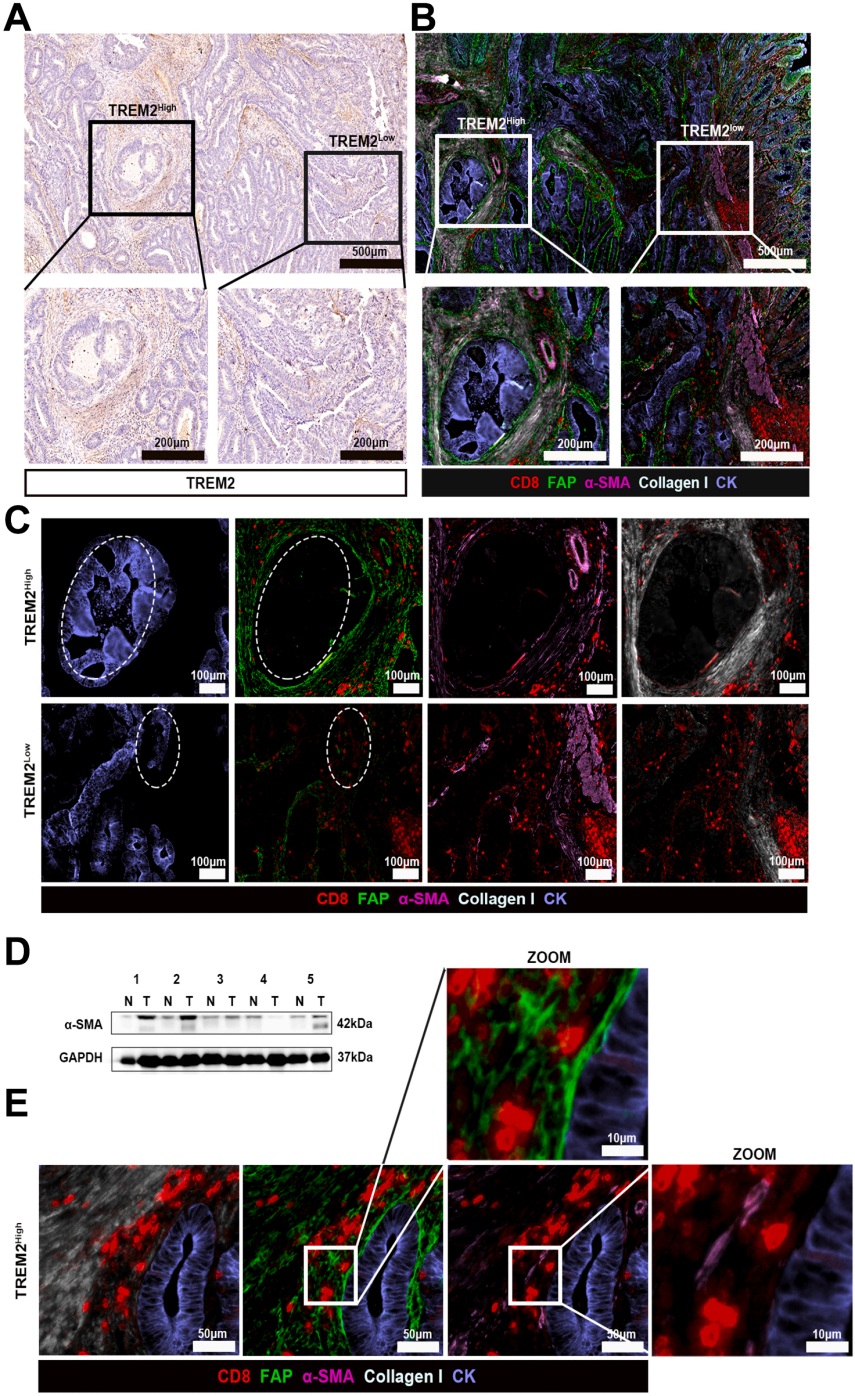
**Inhibition of CD8^+^ T cell infiltration in CRC by FAP^+^ CAFs.** (*A*) Immunohistochemical staining of TREM2 in CRC tissues, depicted in *brown*. (*B* and *C*) Multiplex immunohistochemical staining of CRC tissues for CD8 (*red*), FAP (*green*), α-SMA (*purple*), collagen I (*white*), and cytokeratin (CK; *watchet blue*). (*D*) Western blot analysis of α-SMA protein expression in CRC. N, para-cancer; T, cancer. (*E*) Multiplex immunohistochemical staining of CRC tissues with high TREM2 expression. Scale bars, 500 μm (*upper row* of *A* and *B*), 200 μm (*lower row* of *A* and *B*), 100 μm (*C*), 50 μm (*E*), and 10 μm (*E*, magnified images, marked by *white boxes*).

Previous studies have demonstrated the spatial proximity of α-smooth muscle action (α-SMA)^+^/FAP^+^ CAFs to tumor cells.^10^^,^^11^ To investigate the spatial relationship between CAFs and CD8^+^ T cells, a multi-immunofluorescence staining was conducted for the purpose of an in-depth analysis based on TREM2 immunohistochemical staining on adjacent series sections (Figure 5*A* and *B*). The results showed a higher frequency of FAP^+^ CAFs in regions with higher TREM2 expression. The clustered FAP^+^ CAFs and collagen I formed a barrier that encircled the tumor cells, thereby preventing the CD8^+^ T cells from engaging with them. In contrast, in regions exhibiting diminished TREM2 expression, the sparse FAP^+^ CAFs were unable to form a complete barrier, thereby allowing direct contact between CD8^+^ T cells and tumor cells (Figure 5*C*). The distribution of CAFs expressing α-SMA^+^, a well-established marker for fibroblast activation, was also examined across regions with diverse TREM2 expression levels. Although α-SMA expression was significantly elevated in CRC compared with adjacent noncancerous tissues (Figure 5*D*), α-SMA^+^ CAFs exhibited a scattered distribution throughout the tumor stroma, regardless of the TREM2 expression level, in contrast with the distribution of FAP^+^ CAFs (Figure 5*C*). These results indicate that TREM2 promotes the development of a barrier around tumor cells by stimulating the activity of FAP^+^ CAFs, which results in the confinement of CD8^+^ T cells to the stroma (Figure 5*E*).

### Triggering Receptor Expressed on Myeloid Cells 2–Mediated Activation of Fibroblast-Activated Protein^+^ Cancer-Associated Fibroblasts Is Associated With Transforming Growth Factor-β Signaling

**Figure 6:**
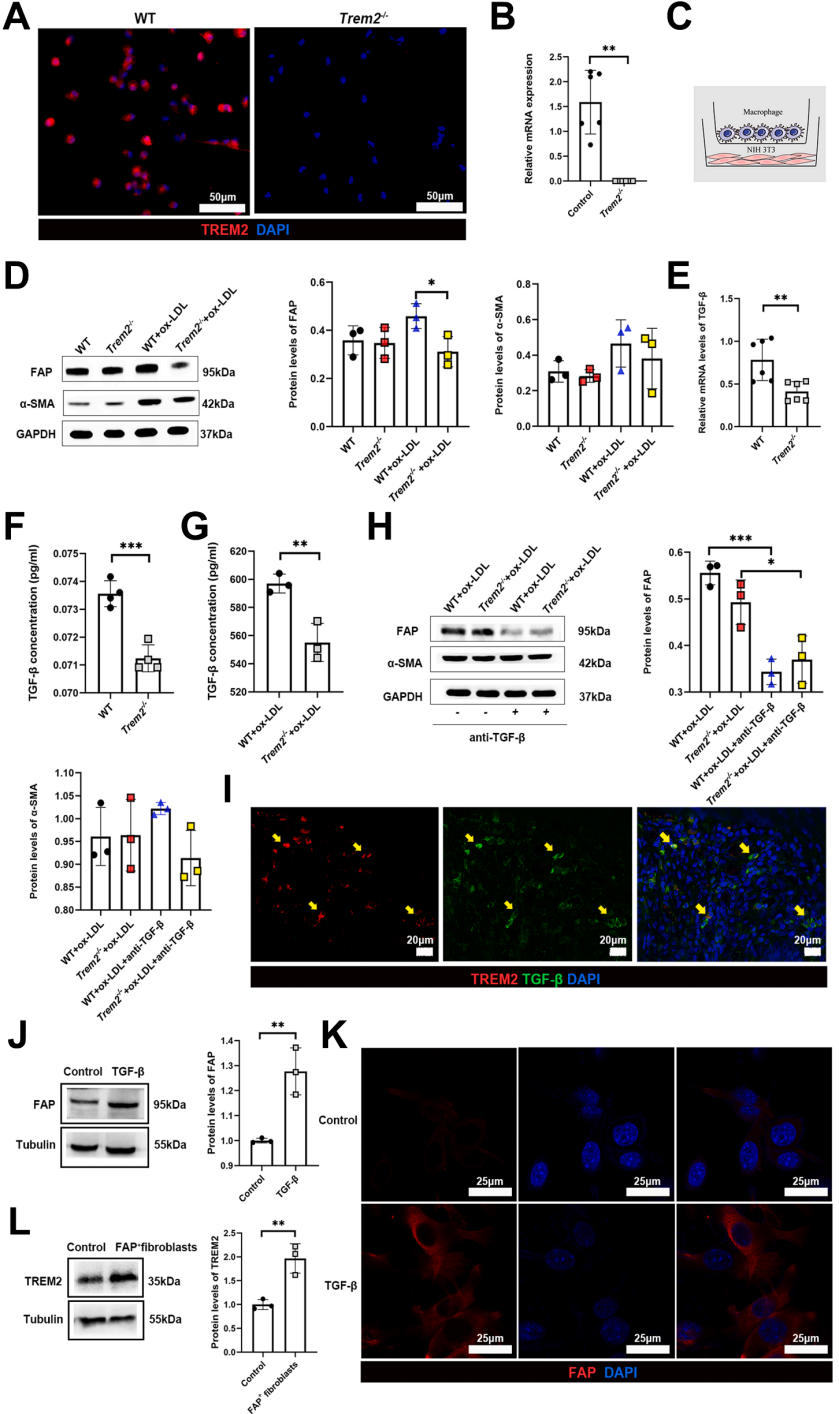
**TREM2-mediated activation of FAP^+^ CAFs via the TGF-β pathway.** (*A*) Immunofluorescence staining of TREM2 in cultured primary macrophages obtained from WT and *Trem2* KO mice. Scale bars, 50 μm. (*B*) TREM2 messenger RNA (mRNA) expression in macrophages. (*C*) Schematic of the experimental strategy for coculturing primary macrophages with NIH3T3 cells for 6 days. (*D*) Western blotting for FAP and α-SMA in NIH3T3 cells. (*E*) TGF-β mRNA expression in macrophages. (*F* and *G*) TGF-β protein concentration in cell culture media. (*H*) Western blotting for FAP and α-SMA in NIH3T3 cells after addition of anti–TGF-β to the coculture system. (*I*) Immunofluorescence staining for TREM2 and TGF-β in CRC tissue. Scale bars, 20 μm. (*J*) Western blotting for FAP in NIH3T3 cells. (*K*) Immunofluorescence staining for FAP in NIH3T3 cells. Scale bars, 25 μm. (*L*) Western blotting for TREM2 in macrophages after addition of FAP^+^fibroblasts to the coculture system. ∗*P* < .05; ∗∗*P* < .01; ∗∗∗*P* < .001. DAPI, 4′,6-diamidino-2-phenylindole.

**Figure 7:**
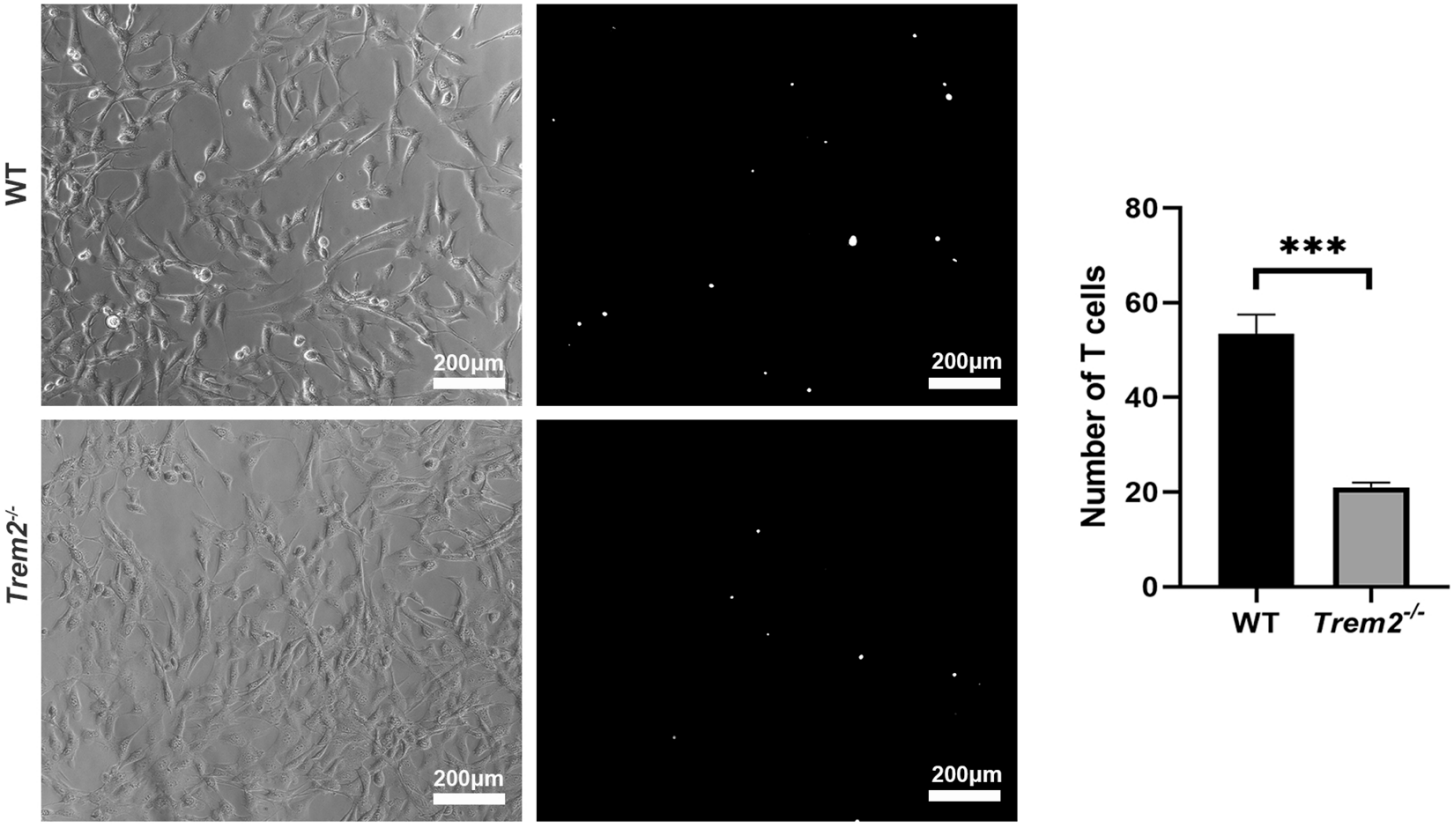
**Involvement of TREM2 in the adhesion between fibroblasts and T cells.** Adhesion assay: Bright-field images (*left*; NIH/3T3 cells and T cells) and fluorescent images (*right*; T cells). ∗∗∗*P* < .001. Scale bars, 200 μm.

The absence of *Trem2* in knockout (KO) mice was confirmed using immunofluorescence staining and quantitative polymerase chain reaction (qPCR) (Figure 6*A* and *B*). Primary macrophages isolated from both wild-type (WT) C57 mice and *Trem2* KO mice were cocultured with NIH3T3 fibroblasts respectively. Macrophage stimulation was achieved by addition of oxidized low-density lipoprotein (ox-LDL) (Figure 6*C*). For the WT macrophages, TREM2 activation triggered upregulation of FAP and α-SMA expression in the cocultured fibroblasts. In contrast, FAP and α-SMA expression in fibroblasts cocultured with *Trem2*^-/-^ macrophages remained largely unaffected, even in the presence of ox-LDL (Figure 6*D*). Considering the well-established role of transforming growth factor-β (TGF-β) in fibroblast differentiation, we utilized qPCR and enzyme-linked immunosorbent assay to evaluate the impact of TREM2 on TGF-β production and release by macrophages. Our results revealed a substantial reduction in both TGF-β synthesis and secretion following deletion of *Trem2* (Figure 6*E–G*). Moreover, the induction of FAP was significantly mitigated by treatment with an anti–TGF-β antibody (Figure 6*H*). Immunofluorescence staining for TREM2 and TGF-β was applied to CRC tissues, showing that the cells expressing TGF-β were primarily TREM2^+^ macrophages (Figure 6*I*). When T cells were introduced into the coculture system, we observed a marked decrease in fibroblast adhesion to T cells in the *Trem2*^-/-^ setting (Figure 7). These results suggest that TREM2 can activate CAFs through the TGF-β signaling pathway, potentially restricting T-cell infiltration.

To address the bidirectionality of the TREM2-FAP axis, the fibroblasts were activated in vitro using recombinant TGF-β (Figure 6*J* and *K*), followed by the coculture with naive macrophages. Compared with the control group, FAP^+^ fibroblasts increased the expression of TREM2 on macrophages (Figure 6*L*). This finding indicates a mutual regulation between TREM2^+^ macrophages and FAP^+^ CAFs, reflecting the complex interplay of the TME.

### Triggering Receptor Expressed on Myeloid Cells 2 Restrains Collagen Degradation by Suppressing Matrix Metalloproteinase 13 Secretion From Macrophages

**Figure 8:**
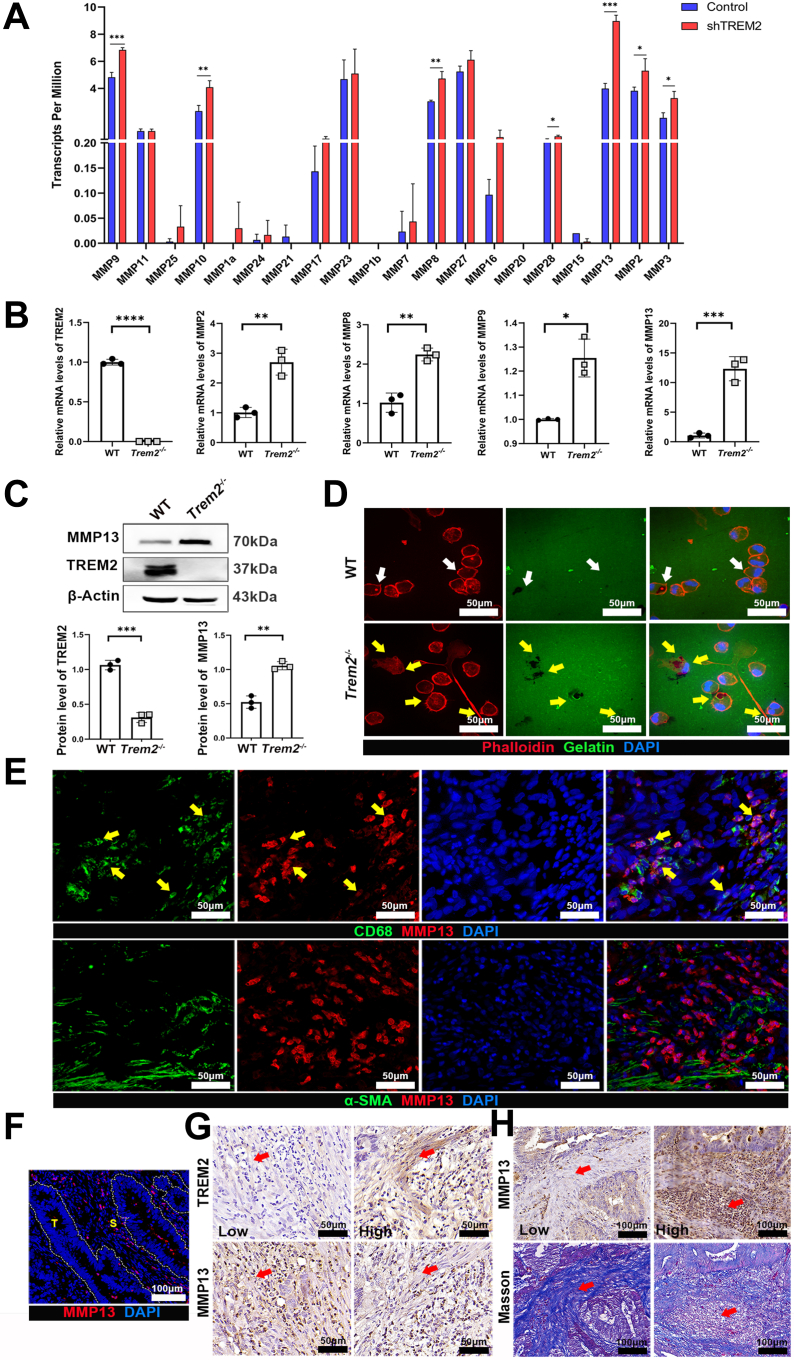
**TREM2 suppression of MMP13 secretion by macrophages to restrict collagen degradation.** (*A*) Transcriptomic profiling of macrophages obtained from WT and *Trem2* KO macrophages. (*B*) Messenger RNA (mRNA) levels of TREM2, MMP2, MMP8, MMP9, and MMP13 in macrophages detected by qPCR. (*C*) Protein expression of TREM2 and MMP13 in macrophages by Western blotting. (*D*) Matrix degradation assay showing enhanced enzymatic activity of *Trem2*^*-/-*^ macrophages. Phalloidin-labeled actin filaments indicate macrophages, and gelatin indicates matrix. (*E*) Dual-immunofluorescence staining for MMP13 with either CD68 or α-SMA. (*F*) Immunofluorescence staining of MMP13 in CRC tissues. S, stroma; T, tumor. (*G*) Immunohistochemical staining of TREM2 and MMP13 in serial sections of CRC tissues. (*H*) MMP13 immunohistochemical staining and Masson’s Trichrome staining in serial sections of CRC tissues. ∗*P* < .05; ∗∗*P* < .01; ∗∗∗*P* < .001; ∗∗∗∗*P* < .0001. Scale bars, 50 μm (*D*, *E* and *G*), 100 μm (*F* and *H*). DAPI, 4′,6-diamidino-2-phenylindole.

*Trem2* was knocked down in macrophages from WT mice using adenovirus, and transcriptomic analysis revealed a significant upregulation of MMPs, enzymes associated with ECM degradation (Figure 8*A*). The messenger RNA expression of matrix metalloproteinase (MMP)2, MMP8, MMP9, and MMP13 in WT and *Trem2*^*-/-*^ macrophages were further validated by qPCR. Consistent with transcriptomic data, these MMPs were significantly upregulated in *Trem2*^*-/-*^ macrophages, with the most pronounced increase observed in MMP13 (Figure 8*B*). Furthermore, MMP13 protein expression was clearly increased in the *Trem2*^*-/-*^ macrophages (Figure 8*C*). In ECM degradation assays, the *Trem2*^*-/-*^ macrophages exhibited enhanced ability to degrade the surrounding matrix, evidenced by increased cavitation around the macrophages (Figure 8*D*). Immunohistochemical analysis of human CRC specimens revealed that MMP13 was predominantly localized to the tumor stroma (Figure 8*F*), particularly in the CD68^+^ macrophages instead of in the α-SMA^+^ fibroblasts (Figure 8*E*). Notably, MMP13 expression was decreased in the stromal region with high TREM2 expression and elevated in those with low TREM2 expression (Figure 8*G*). Similarly, TREM2 expression exhibited an inverse correlation with collagen content (Figure 8*H*). Collectively, these data suggest that the elevated TREM2 inhibits MMP13 secretion by macrophages, consequently restricting ECM degradation.

**Figure 9:**
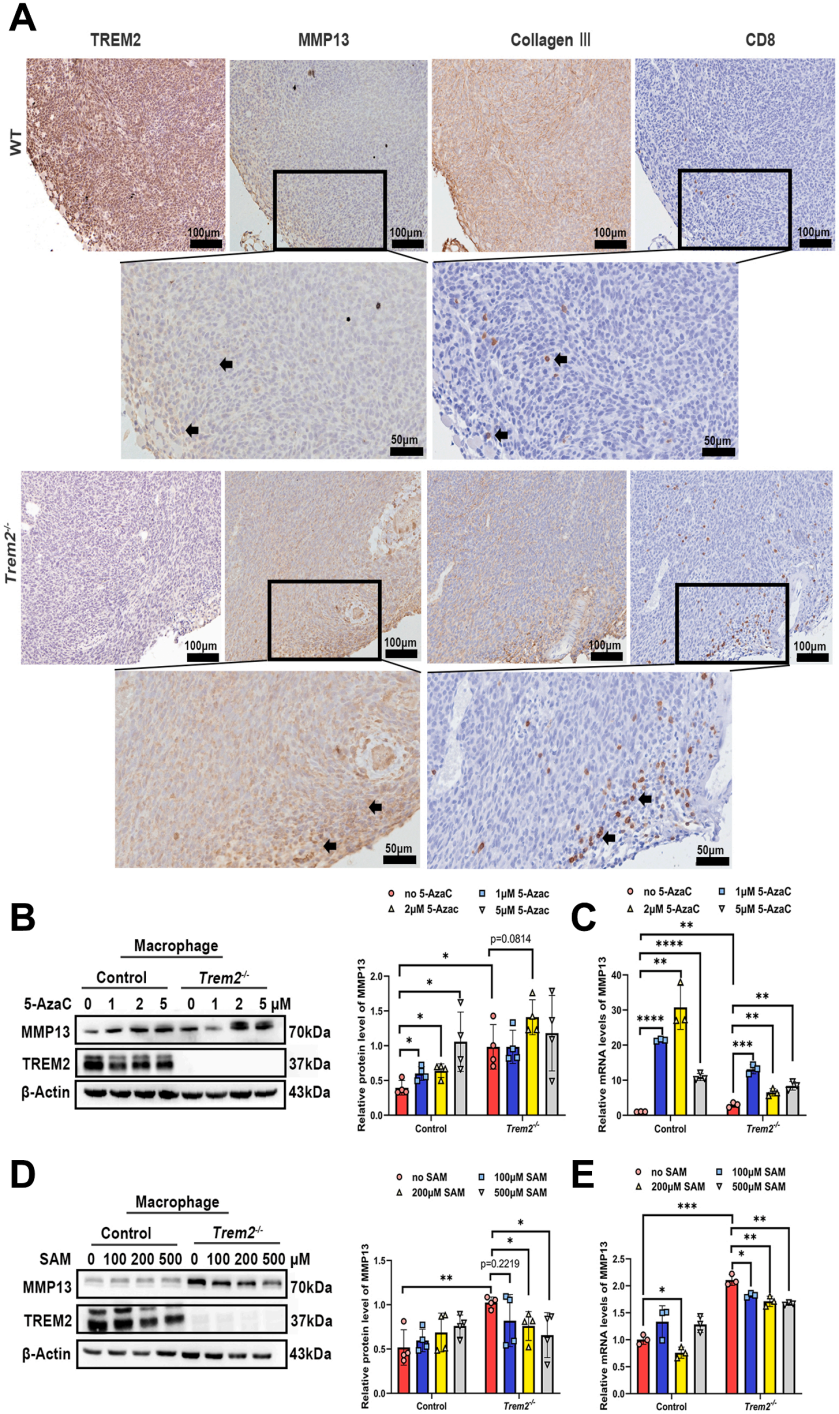
**TREM2 suppression of MMP13 secretion by macrophages to restrict collagen degradation.** (*A*) Immunohistochemical staining for TREM2, MMP13, Collagen III and CD8 expression in tumor. Scale bars, 100 μm and 50 μm (magnified images, marked by *black boxes*). (*B* and *D*) Western blotting for TREM2 and MMP13 in macrophages. (*C* and *E*) Messenger RNA (mRNA) levels of MMP13 in macrophages detected by qPCR. ∗*P* < .05; ∗∗*P* < .01; ∗∗∗*P* < .001; ∗∗∗∗*P* < .0001. 5-AzaC, 5-azacytidine; SAM, S-adenosylmethionine.

To investigate the in vivo role of TREM2, a tumor-bearing mouse model was established in WT and *Trem2*^*-/-*^ mice. Immunohistochemical staining revealed the expression and distribution patterns of TREM2, MMP13, FAP, and CD8 within tumor tissues. Compared with WT mice, *Trem2*^*-/-*^ mice exhibited increased MMP13 expression, more extensive collagen degradation, and enhanced CD8^+^ T cell infiltration (Figure 9*A*). Emerging evidence indicates that MMP13 expression is regulated by DNA methylation.^12^^,^^13^ We examined whether this epigenetic mechanism underlies TREM2-mediated regulation. Treatment of macrophages with 5-azacytidine (a DNA methylation inhibitor) or S-adenosylmethionine (a methyl donor) modulated MMP13 expression in a manner consistent with methylation-dependent control (Figure 9*B–E*). The results indicate that DNA methylation may serve as a potential mechanism underlying TREM2-mediated regulation of MMP13 expression in macrophages.

### Triggering Receptor Expressed on Myeloid Cells 2–Mediated Activation of Cancer-Associated Fibroblasts Promotes Immune Escape

**Figure 10:**
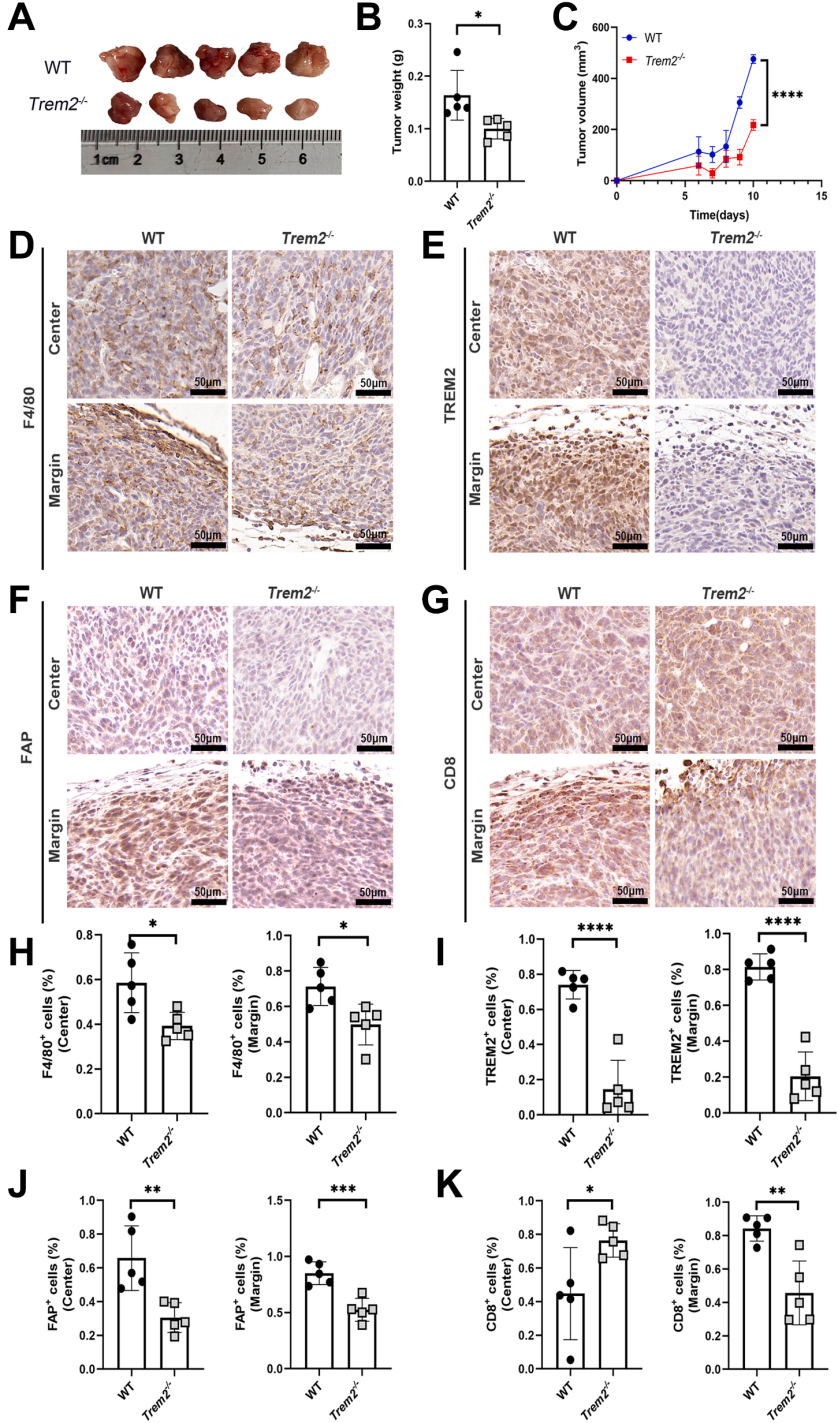
**TREM2-mediated activation of CAFs facilitates immune escape.** A tumor-bearing mouse model was established using MC38 cells. (*A*) Gross image showing subcutaneous xenograft tumor model (n = 5). (*B* and *C*) Tumor volume and weight at 10 days post-tumor grafting. (*D–K*) Immunohistochemical staining and statistical analysis for F4/80, TREM2, FAP, and CD8 expression in the tumor center and margin. ∗*P* < .05; ∗∗*P* < .01; ∗∗∗*P* < .001; ∗∗∗∗*P* < .0001. Scale bars, 50 μm (*D–G*).

**Figure 11:**
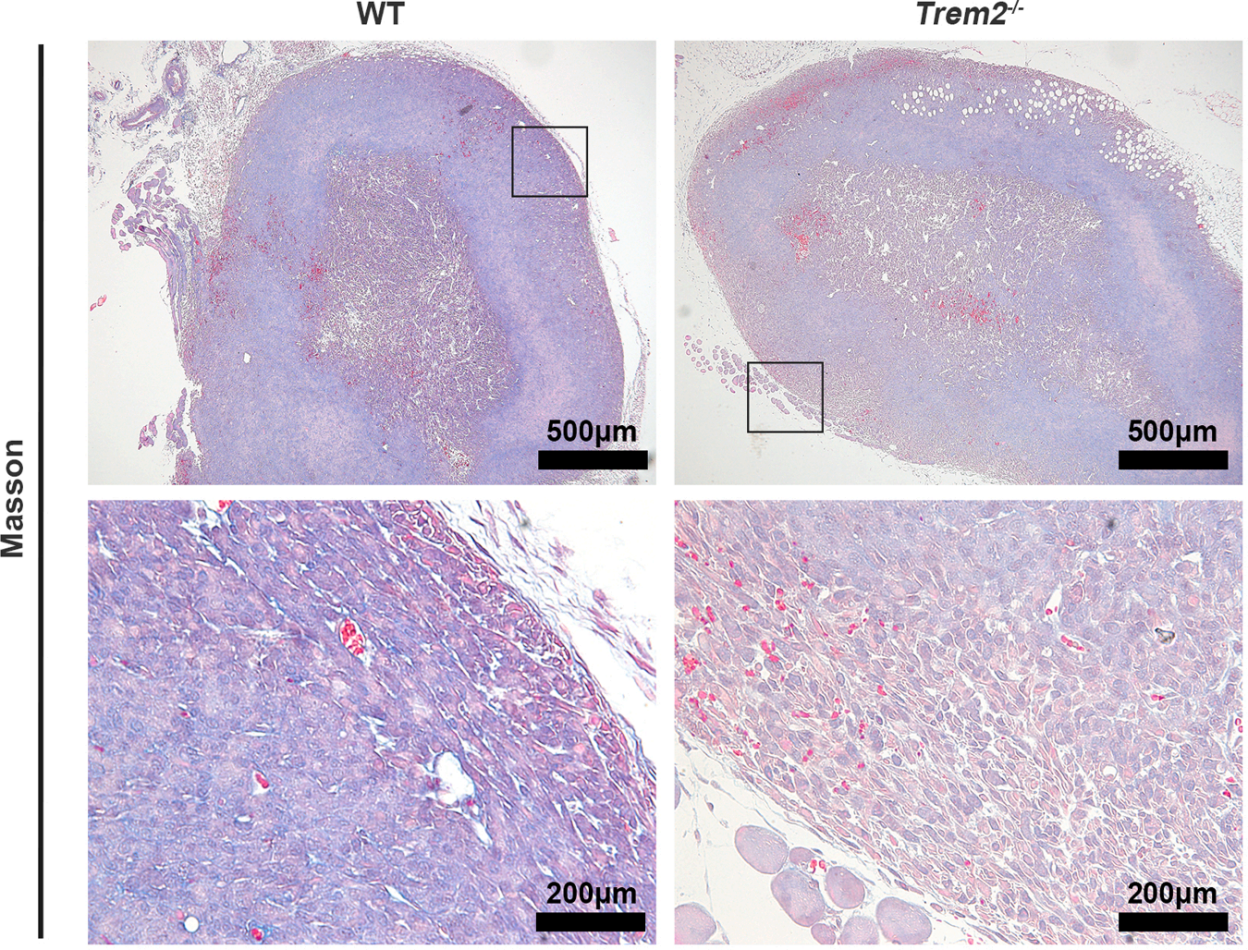
**A tumor-bearing mouse model was established using MC38 cells.** Masson’s Trichrome staining of mouse tumor tissues. Scale bars, 500 μm (*upper row*), 200 μm (*lower row*).

**Figure 12:**
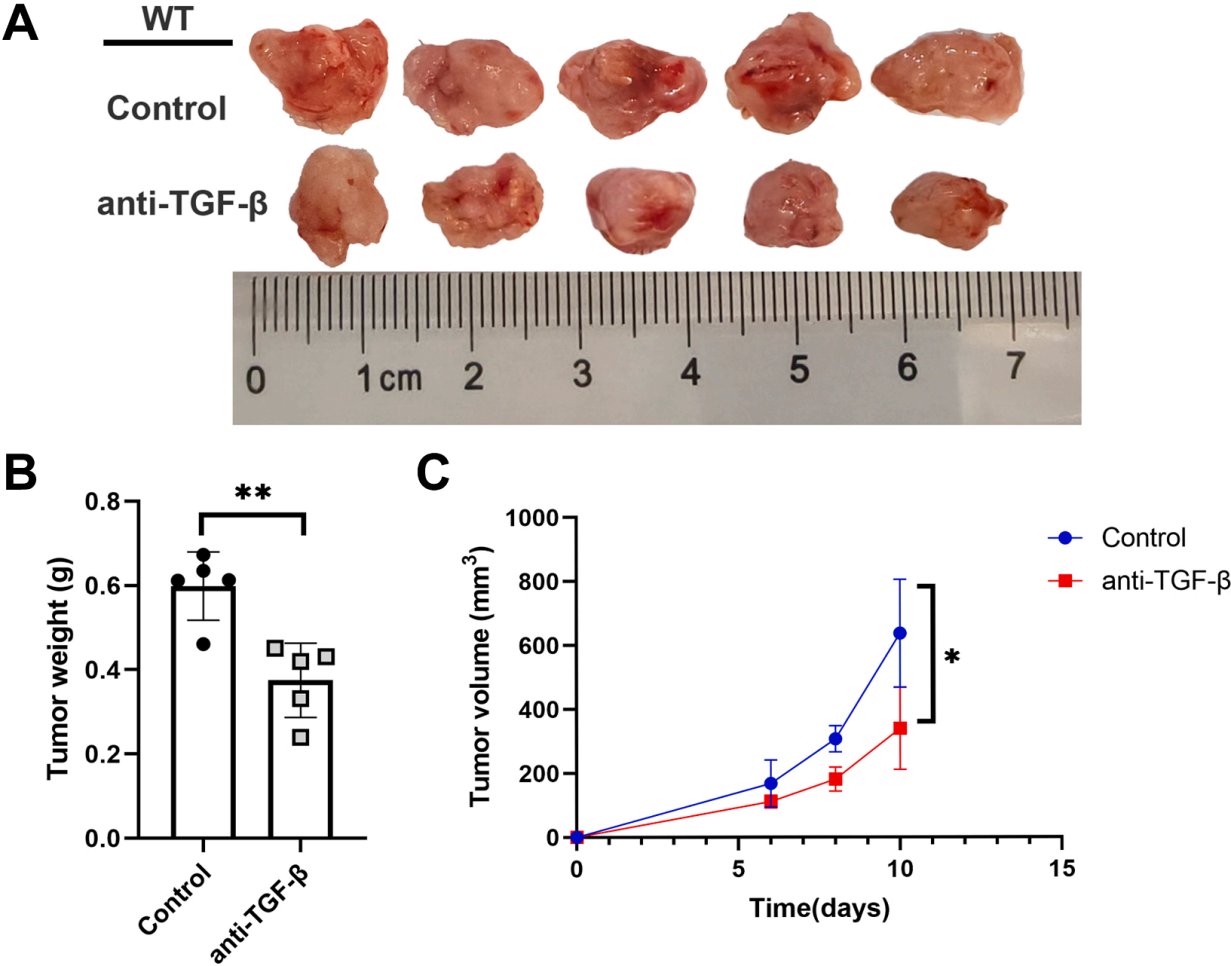
**TGF-β neutralization blockade experiments in CRC mouse model.** A tumor-bearing mouse model was established using MC38 cells. (*A*) Gross image showing subcutaneous xenograft tumor model (n = 5). (*B* and *C*) Tumor volume and weight at 10 days post-tumor grafting. ∗*P* < .05; ∗∗*P* < .01.

**Figure 13:**
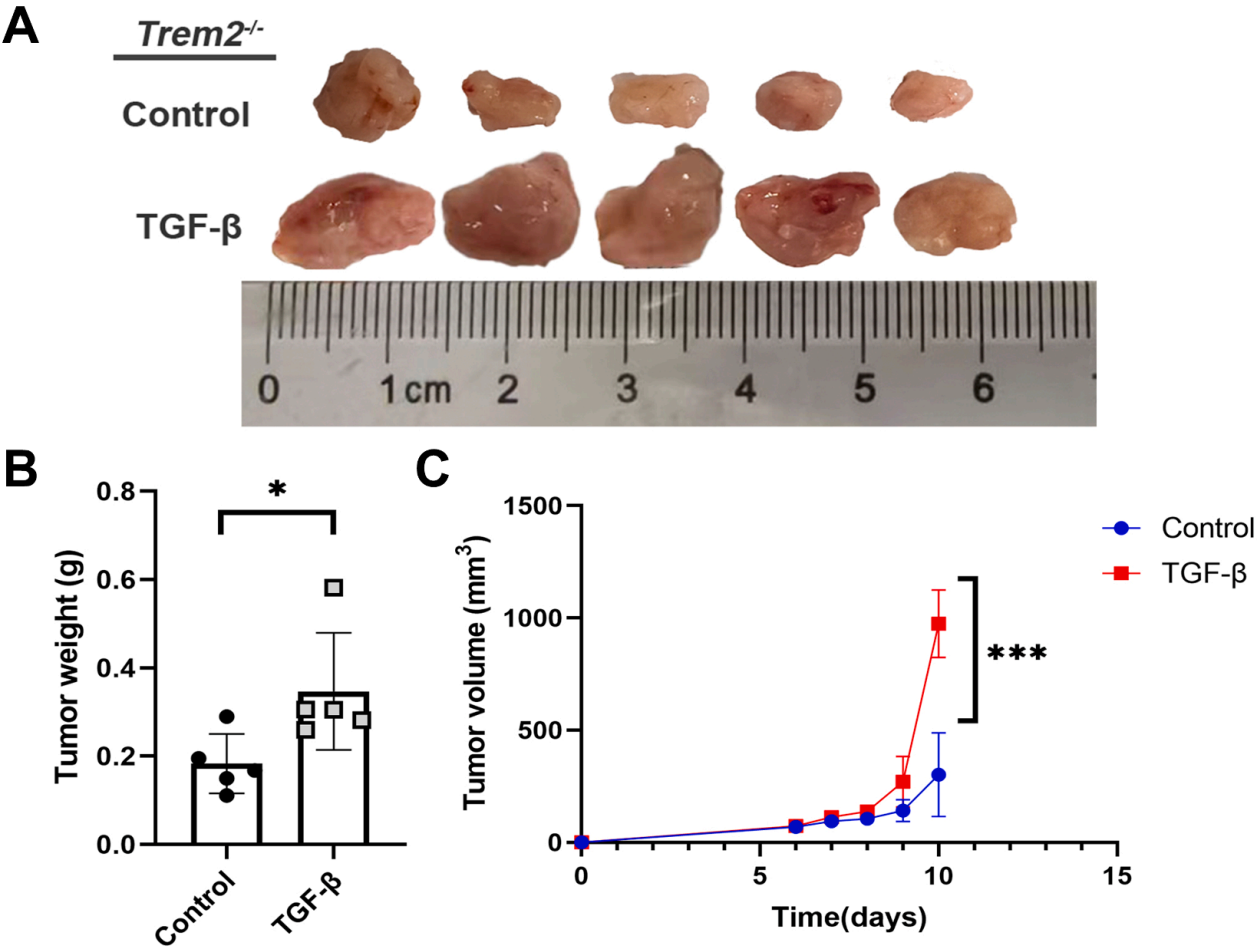
**Rescue experiments via exogenous TGF-β administration in *Trem2*^*-/-*^ mice.** A tumor-bearing mouse model was established using MC38 cells. (*A*) Gross image showing subcutaneous xenograft tumor model (n = 5). (*B* and *C*) Tumor volume and weight at 10 days post-tumor grafting. ∗*P* < .05; ∗∗∗*P* < .001.

**Figure 14:**
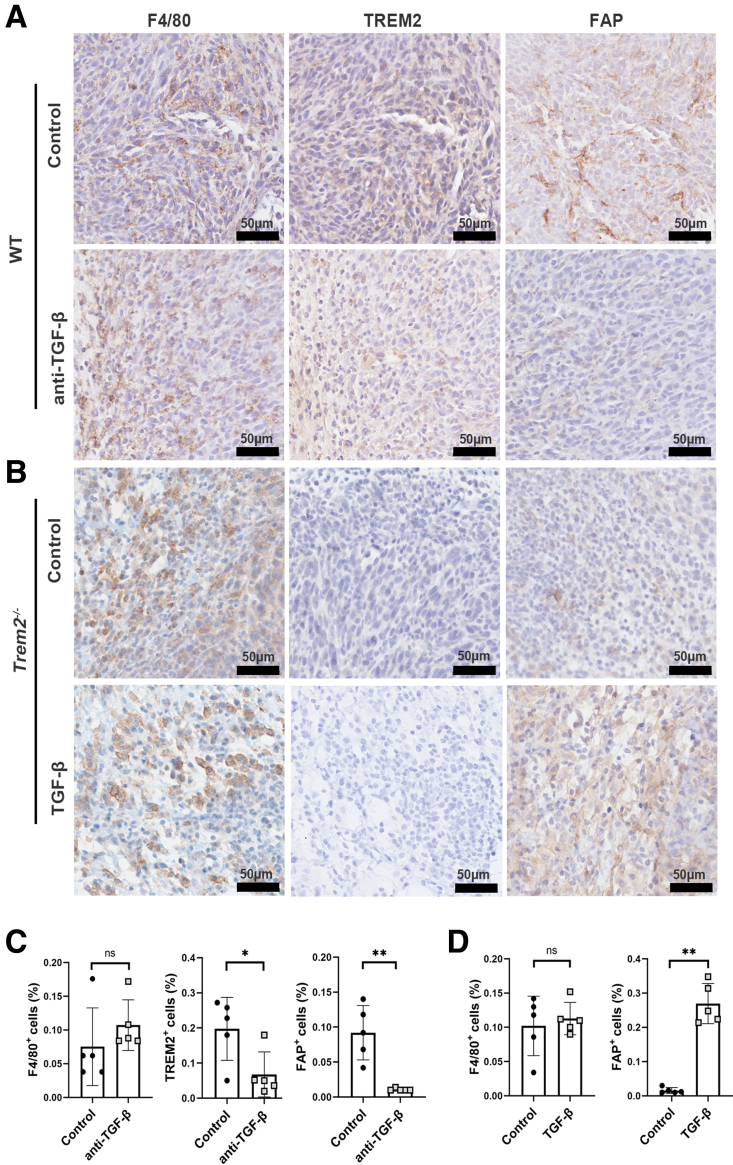
**TGF-β neutralization blockade experiments in CRC mouse model and rescue experiments via exogenous TGF-β administration in *Trem2^-/-^*mice.** (*A–D*) Immunohistochemical staining and statistical analysis for F4/80, TREM2, and FAP expression in tumor. ∗*P* < .05; ∗∗*P* < .01. Scale bars, 50 μm (*A and B*).

The weight and volume of the grafted tumors were significantly reduced in *Trem2*^*-/-*^ mice compared with those of the WT mice at 10 days post-tumor grafting (Figure 10*A–C*). Immunohistochemical staining of tumor tissues revealed markedly lower expression of F4/80 (Figure 10*D* and *H*), TREM2 (Figure 10*E* and *I*), and FAP (Figure 10*F* and *J*) in both the center and margin of tumors from *Trem2*^*-/-*^ mice relative to WT mice. In WT tumors, CD8^+^ T cells were largely confined to the collagen-rich tumor margin (Figure 11), with minimal infiltration into the tumor center (Figure 10*G* and *K*). In contrast, *Trem2*^*-/-*^ tumors exhibited substantially increased CD8^+^ T cell infiltration into the central region (Figure 10*G* and *K*). To directly corroborate the role of the TREM2-TGF-β-CAF pathway in vivo, we performed TGF-β neutralization blockade experiments in a CRC mouse model and rescue experiments via exogenous TGF-β administration in *Trem2*^*-/-*^ mice (Figures 12*A–C* and 13*A–C*). Neutralizing TGF-β significantly reduced FAP expression compared with the control group. This finding is consistent with our previous in vitro coculture results (Figure 6*H*). Conversely, exogenous TGF-β treatment restored FAP expression in *Trem2*^*-/-*^ mice (Figure 14*A–D*). Collectively, these data indicate that TREM2-mediated CAF activation leads to collagen fiber deposition, forming a barrier that restricts the infiltration of CD8^+^ T cells into the tumor parenchyma. This mechanism ultimately facilitates tumor immune escape.

## Discussion

Cancer immunotherapy has provided significant advancements in hematologic malignancies but has shown limited promise in solid tumor treatment. This discrepancy is largely attributed to immune escape and immunosuppressive mechanisms in the TME that remain poorly understood.^5^ The complexity of immunosuppressive mechanisms relates to the functionality, quantity, and distribution of CD8^+^ T cells.^14^^,^^9^ The complex TME is also known to be instrumental in tumor progression.^15^ Within the tumor stroma, fibrous stromal components are particularly noted for their contribution to the drug resistance of solid tumors.^7^ Recent studies have highlighted the role of CAFs within the TME.^16^, ^17^, ^18^, ^19^, ^20^ These cells can secrete collagen to form a fibrous barrier that impedes the infiltration of immune cells into the tumor parenchyma, ultimately impacting the effectiveness of both antitumor immunity and antitumor therapies.^14^^,^^9^ FAP is a surface protein that is upregulated in several types of cancers and serves as a marker for activated CAFs. FAP^+^ CAFs secrete collagen, cytokines, and other factors that facilitate tumor stroma remodeling and the creation of an immunosuppressive microenvironment. However, the precise mechanisms that trigger the activation of FAP^+^ fibroblasts remain incompletely understood.^21^^,^^22^ In the present study, we uncovered a major reason for the inability of CD8^+^ T cells to engage with CRC cells in the CRC microenvironment, namely, the activation of FAP^+^ CAFs by TREM2, which leads to the formation of fibrous barrier structures that impede infiltration of CD8^+^ T cells. Through multiple immunohistochemical staining experiments, we identified FAP^+^ CAFs as a predominant CAF subtype in the CRC stroma that is densely arranged around the tumor cells. The collagen in the stroma was distributed in multilayered sheets, which also posed a challenge for the migratory movement of CD8^+^ T cells.

Previous studies of TREM2 have predominantly focused on its roles in the nervous system, including the development and progression of Alzheimer’s disease. Recently, an increasing number of studies have demonstrated elevated TREM2 expression in various tumor types.^23^ However, the localization and function of TREM2 vary across different types of tumors. In some tumors, TREM2 expression is negatively correlated with the infiltration of most immune cells but positively correlated with the infiltration TAMs.^8^ Another study found that TREM2^+^ macrophages are able to cause immunosuppression by inhibiting cytotoxic T-cell proliferation and recruiting forkhead box P3^+^ regulatory T cells into the TME.^24^^,^^25^ The regulatory role of TREM2^+^ TAMs in relation to CAFs remains unclear. Our study delved into the activation of CAFs by TREM2^+^ TAMs, and the results enhance our understanding of the interplay between TAMs and the immune escape of tumor cells.

Previous studies have reported an interactive relationship between TAMs and CAFs. TAMs activate CAFs by releasing TGF-β, fibroblast growth factor, and platelet-derived growth factor. Conversely, macrophage recruitment is supported by CAF-derived factors such as periostin, interleukin6, stromal cell-derived factor 1, macrophage colony-stimulating factor, C-C motif ligand 2, and chitinase-3-like protein 1. This bidirectional communication establishes a regulatory loop between the 2 cell types.^26^, ^27^, ^28^, ^29^ Our results indicate that TGF-β secreted by TAMs with elevated TREM2 expression is a principal molecule responsible for CAF activation. Although various cells, such as tumor cells, are known to secrete TGF-β,^30^ our immunohistochemistry analysis revealed that the concentration of TGF-β was significantly higher within the tumor stroma than at the tumor cells, with TREM2-expressing TAMs identified as the primary source of TGF-β. In recent years, overexpression of MMP13 has been observed in a variety of cancers, including breast cancer, head and neck cancer, and multiple myeloma. Elevated levels of MMP13 are typically correlated with tumor invasion, unfavorable prognosis, and diminished patient survival.^31^ Within the context of CRC, MMP13 is expressed in the cytoplasm of cell lines.^32^^,^^33^ However, our research identified that in CRC tissues, MMP13 was mainly expressed by macrophages in the tumor stroma, with minimal expression in tumor cells and fibroblasts.

CAFs exhibit a high degree of heterogeneity, stemming from the diverse origins of fibroblasts within the TME, including tissue-resident fibroblasts, bone marrow–derived mesenchymal stem cells, epithelial cells, and endothelial cells. Numerous studies have demonstrated that α-SMA^+^ CAFs exert immunosuppressive effects, such as facilitating epithelial–mesenchymal transition processes,^34^ impeding the infiltration of cytotoxic lymphocytes into tumors,^35^ inducing elevated programmed death ligand 1 expression in lung cancer tumor cells,^36^ and contributing to poor prognosis in gastric cancer.^37^^,^^38^ In our study, we identified FAP^+^ CAFs, predominantly modulated by TREM2^+^ macrophages, as a predominant subtype within the CRC stroma. In our experiments, the FAP^+^ CAFs constituted a significant element of the fibrous barrier encircling CRC cells, effectively restricting the infiltration and interaction of T cells with the tumor cells. These findings indicate that targeting TREM2 to modulate CAF activation may offer a novel strategy for cancer immunotherapy and support further research to identify reliable targets for therapeutic intervention.

## Materials and Methods

### Colorectal Cancer Tissue Acquisition

All CRC specimens and adjacent normal mucosal tissues were collected from patients with CRC who had not undergone preoperative chemotherapy or radiotherapy and who did undergo surgery at the General Hospital of Ningxia Medical University between April and September 2020. All biopsy specimens were reviewed by a pathologist and diagnosed as CRC. Tissue samples were obtained with informed, written consent from patients and approval from the Clinical Research Ethics Committee of General Hospital of Ningxia Medical University. The patient information is listed in Table 1.

**Table 1:** Clinical and Pathologic Characteristics of the Patient Cohort

Characteristic	CRC and its adjacent tissues
Sex	Male (n = 19; 61.3%) and female (n = 12; 38.7%)
Age, *y*	Median, 65; range, 37–80
Diagnosis	All patients were histologically confirmed with colorectal adenocarcinoma, and 32.3% of cases had lymph node metastasis

CRC, colorectal cancer.

### Animals and Cell Lines

C57BL/6J mice, aged 6 to 8 weeks, were purchased from the Animal Center of Capital Medical University. *Trem2* KO mice of the same age were purchased from Cyagen Biosciences. *Trem2* KO mice were generated using the CRISPR/Cas9-mediated gene editing technique. All animals were housed under specific pathogen-free conditions in accordance with standard protocols. The animal experiments were approved by Capital Medical University’s Institutional Animal Care and Ethics Committee (Animal Ethical Approval Number: AEEI-2021-003). The mouse colon carcinoma cell line MC38 and mouse NIH3T3 fibroblasts were cultured in Dulbecco’s Modified Eagle’s Medium (DMEM; Gibco) supplemented with 10% fetal bovine serum (Biological Industries), 100 U/mL of penicillin-streptomycin (Gibco), and 1% of mycoplasma removal agent Myco-3 (AppliChem).

### Ethics Approval

The study was conducted according to the guidelines of the Declaration of Helsinki, and approved by the Institutional Review Board of the Capital Medical University.

### Isolation and Transfection of Primary Peritoneal Macrophages and Primary Lung Fibroblasts

Mice were humanely euthanized via intraperitoneal injection of 4% sterile mercaptoacetate solution (Sigma). Subsequently, the peritoneal cavity was lavaged with sterile saline solution. The extracted fluid, containing the macrophages, was subjected to a low-speed centrifugation process to pellet the cells. The cell pellet was resuspended in DMEM and added to culture plates. After a period of 4 hours for adhesion, the culture medium was refreshed to remove nonadherent cells, thus leaving behind the primary peritoneal macrophages, which were then prepared for further experimental procedures. TREM2 adeno-associated virus and its control virus were designed and manufactured by Hanheng Bioengineering Co. Primary macrophages were infected with adeno-associated virus as directed in the manual. In brief, 1 mL of cell suspension containing 5 × 10^5^ cells was added to a well of a 24-well plate. After 24 hours, the corresponding amount of virus was added based on a multiplicity of infection of 20. Sixteen hours after the virus infection, the culture medium was replaced. The infection effect was observed under a fluorescence microscope 96 hours later and the expression of TREM2 was detected.

Following euthanasia, mouse lung tissue was extracted for the purpose of obtaining lung primary fibroblasts. These fibroblasts were cultured in DMEM supplemented with 10% fetal bovine serum. All cells were incubated in a humidified condition at 37 °C with 5% CO_2._

### Tumor-Bearing Mouse Model

MC38 cells and NIH3T3 fibroblasts or mouse lung fibroblasts (in a ratio of 1:3, totaling 1 × 10^6^ cells) were suspended in a mixture of 100 μL phosphate-buffered saline (PBS) and subcutaneously injected into the right axilla of mice. Tumor diameters were measured daily with vernier calipers once the tumors became visible. Tumor volumes were calculated using the standard formula: V = 0.52 × L × Sˆ2 (where L is the long diameter and S is the short diameter). On the 11th day postinoculation, when the tumor diameters reached 7–10 mm, the mice were euthanized, and all tumors were excised, measured, and weighed.

#### Rescue experiments via exogenous transforming growth factor-β administration in Trem2^-/-^ mice

The tumor-bearing mice were treated with TGF-β (Novoprotein, Catalog C16W) at a dose of 3 μg/kg via intraperitoneal injection once every 48 hours.

#### Transforming growth factor-β neutralization blockade experiments in the colorectal cancer mouse model

The tumor-bearing mice were treated with anti–TGF-β (BioXcell, Catalog BE0057) at a dose of 10 mg/kg via intraperitoneal injection once every 72 hours.

### Western Blot Analysis

Total proteins were extracted using radioimmunoprecipitation assay lysis buffer (Applygen) containing protease inhibitors (Sigma-Aldrich) and phosphatase inhibitors (Sigma-Aldrich). Protein concentration was measured using a bicinchoninic acid protein assay kit. The proteins were subjected to 10% sodium dodecyl sulfate-polyacrylamide gel electrophoresis and transferred to polyvinylidene difluoride membranes (Millipore). After blocking, the membranes were incubated with the primary antibody overnight at 4 °C (TREM2; Cell Signaling Technology, Catalog #91068; TREM2; R&D, Catalog AF1729; TREM2; Abclonal, Catalog A22134; FAP; BOSTER, Catalog BM5121; α-SMA; Abcam, Catalog ab5694; MMP13; Proteintech, Catalog 18165-1-AP; β-Actin (C4); SANTA, Catalog sc-47778; glyceraldehyde 3-phosphate dehydrogenase; Cell Signaling Technology, Catalog 2118S), followed by incubation with corresponding horseradish peroxidase-conjugated secondary antibodies for 1 hour at room temperature. The immunoblotting bands were detected using an enhanced chemiluminescence reagent (Thermo Fisher Scientific) on a Fusion FX Vilber Lourmat (France).

### Masson’s Trichrome Stain

The tissues were formalin-fixed, paraffin-embedded, and cut into 5-μm sections. Sections were routinely deparaffinized to aqueous solution, stained with a Masson’s Staining Trichrome Kit (Solarbio, Catalog #G1340), and then sealed with neutral balsam. The staining results were observed using Slide Viewer software.

### Immunohistochemistry

The formalin-fixed and paraffin-embedded tissues were cut into 5-μm sections. Sections were placed in an oven at 60 °C for 1 hour, then routinely deparaffinized for 1 hour, and allowed to flatten in a 92 °C to 98 °C water bath before blocking of endogenous peroxidase. Primary antibody solution was added dropwise before incubation overnight at 4 °C (TREM2; Cell Signaling Technology, Catalog #91068; FAP; BOSTER, Catalog BM5121; F4/80/Adgre1; BOSTER, Catalog A08751; MMP13; Proteintech, Catalog 18165-1-AP; CD8; ThermoFisher, Catalog #MA5-14548; Collagen I; Abcam, Catalog ab34710; CK; Abcarta Medtech, Catalog PA125), followed by exposure to the respective secondary antibody. Staining was observed under a microscope and statistically analyzed.

### Immunofluorescence Staining

The paraffin-embedded sections underwent standard rehydration, antigen retrieval, and blocking prior to overnight incubation with a specific primary antibody (TREM2; Cell Signaling Technology, Catalog #91068; CD68; Abcam, Catalog Ab955; Collagen I; Abcam, Catalog ab34710). Subsequently, the sections were incubated with a secondary antibody and observed under a fluorescence microscope.

### Cell Coculture and Cell Adhesion Assay

Mouse peritoneal primary macrophages were cocultured with mouse fibroblast cell line NIH3T3 using the Transwell coculture system (Corning, Catalog #3414). The macrophages were cultured in the upper chamber, whereas NIH3T3 cells were maintained in the lower chamber. Macrophages were stimulated by the addition of ox-LDL (Solarbio, Catalog #IO1300) to the upper chamber. Following 6 days of coculture, total protein was extracted for subsequent immunoblot analysis. For the cell adhesion assay, the upper chamber was removed after 3 days of coculture, and mouse spleen T cells stained with mito-red (Dojindo, Catalog# R237) were then added to the lower chamber. After 4 hours of incubation, the culture medium was removed, and the cells were gently washed twice with PBS before the adhesion of T cells was observed on a fluorescence microscope. NIH 3T3 cells were activated in vitro with 10 ng/mL recombinant TGF-β. After 24 hours, FAP expression was assessed. These activated fibroblasts were then cocultured with macrophages, and TREM2 expression levels were evaluated after 48 hours.

### Reverse Transcription-Quantitative Polymerase Chain Reaction

Total RNA was extracted using TRIzol reagent (Invitrogen, Catalog #15596026), according to the manufacturer’s instructions. RNA was reverse-transcribed to complementary DNA using 5× All-In-One RT Master Mix (ABM). qPCR was performed using SYBR Premix Ex Taq Kit (Applied Biosystems) and detected on the ABI7500 qPCR instrument (Applied Biosystems). The nucleotide sequences of all primers are listed in Table 2.

**Table 2:** The Nucleotide Sequences of All Primers

Gene	Sequence (5′-3′)
TGFb1 forward primer	ACTGGAGTTGTACGGCAGTG
TGFb1 reverse primer	GGGGCTGATCCCGTTGATTT
CXCL12 forward primer	CAACACTCCAAACTGTGCCC
CXCL12 reverse primer	CCTTTGGGCTGTTGTGCTTAC
CX3CL1 forward primer	TGCCCTGACAAAGCCTGAAT
CX3CL1 reverse primer	CATCCTGTGCCTCGGAAGTT
CXCL10 forward primer	GAAGGTCAGCACACGGTTTC
CXCL10 reverse primer	CTCCTTGCCTGAGCCTAACC
CXCL9 forward primer	CGAGGCACGATCCACTACAA
CXCL9 reverse primer	GAGTCCGGATCTAGGCAGGT
CCL2 forward primer	CACTCACCTGCTGCTACTCA
CCL2 reverse primer	GGTCAGCACAGACCTCTCTC
TREM2 forward primer	ACAGCACCTCCAGGAATCAAG
TREM2 reverse primer	ACTTGCTCAGGAGAACGCAG
MMP13 forward primer	TCACCTGATTCTTGCGTGCT
MMP13 reverse primer	AGCAGATGGACCCCATGTTT
GAPDH forward primer	TGCACCACCAACTGCTTAG
GAPDH reverse primer	GGATGCAGGGATGATGTTC

### Extracellular Matrix Degradation

Gelatin from pig skin with conjugated fluorescein (Thermo Fisher, G13187) was diluted to 1 mg/mL in PBS containing 2% sucrose (Solarbio, S8271) and subsequently diluted to 0.2 mg/mL with distilled water. The gelatin working solution was applied to a cell culture coverslip and positioned within the wells of a 24-well plate, prior to the addition of cold 0.5% glutaraldehyde solution for fixation. After removal of the glutaraldehyde solution, the sodium borohydride solution was added and left for 3 minutes before removal. Cells were inoculated on the gelatin and fixed in 4% formaldehyde solution for 30 minutes. Phalloidin (rhodamine phalloidin; Cytoskeleton) was then added and incubated for 30 minutes under a confocal microscope.

### Enzyme-Linked Immunosorbent Assay

Cell culture supernatants were collected from mouse peritoneal primary macrophages cocultured with the NIH3T3 mouse fibroblast cell line for 6 days. The supernatants were immediately placed on ice to stabilize protein integrity. The impact of TREM2 on TGF-β production and release by macrophages were evaluated using the Mouse TGF-β 1 ELISA Kit (RayBiotech, Catalog #ELM-TGFb1).

### Statistical Analysis

Data are expressed as mean ± standard deviation. Statistical analyses were performed using the unpaired Student *t* test, analysis of variance, and the Kaplan–Meier method as appropriate. Values of *P* ≤ .05 indicated statistical significance.
